# New views of hypnotizability

**DOI:** 10.3389/fnbeh.2014.00224

**Published:** 2014-06-24

**Authors:** Enrica L. Santarcangelo

**Affiliations:** Department of Translational Research and New Technologies in Medicine and Surgery, University of PisaPisa, Italy

**Keywords:** hypnotizability, imagery, cerebellum, postural control, voluntariness, nitric oxide

## Introduction

Hypnotizability, or hypnotic susceptibility (Green et al., 2005), is a good predictor of the response to suggestions in and out of hypnosis (Meyer and Lynn, 2011). It can be measured by scales (Sheehan and McConkey, 1982) allowing to classify subjects as high (*highs*), medium (*mediums*), and low (*lows*) hypnotizable by indicating the individual ability to modify experience and behavior according to the suggestions' content and to feel that this occurs independently of will.

**Figure 1:**
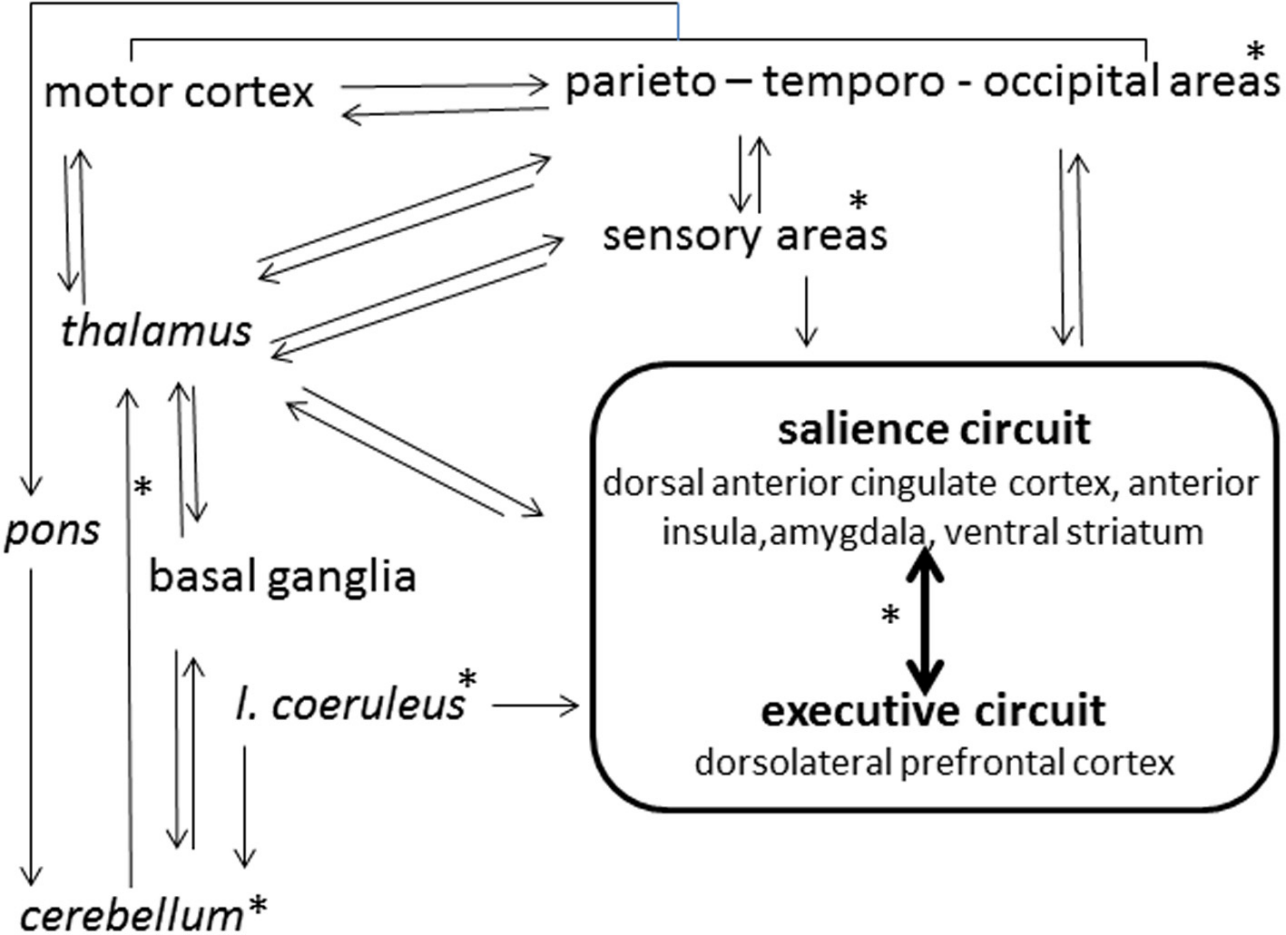
**Schematic view of hypnotizability-related differences in the brain activity**. ^*^Structures and connections (arrows) possibly involved.

In the ordinary state of consciousness, *highs* and *lows* can be discriminated by the predictability of their electroencephalographic (EEG) dynamics, while the several studies performed through spectral analysis of EEG failed to indicate clear-cut discrimination criteria (Madeo et al., 2013). Imaging studies (Hoeft et al., 2012) have associated high hypnotizability with greater functional connectivity between the left dorsolateral prefrontal cortex, an executive-control region, and the salience network (dorsal anterior cingulate cortex, anterior insula, amygdala, ventral striatum) involved in detection and processing of relevant information (Figure 1). This is consistent with the evidence that *highs* tend to become deeply absorbed in any task of everyday life (Tellegen and Atkinson, 1974; Kihlstrom et al., 1989); nonetheless, the theory attributing the *highs'* peculiar ability of focusing attention on selected internal or external objects as the basis of hypnotic responding (Raz, 2005; Szekely et al., 2010) has been challenged by neuropsychological and genetic studies. The former have denied any association between various attentional abilities and hypnotisability (Varga et al., 2011), have shown only higher arousal in *highs* (Castellani et al., 2007) and have suggested that the *highs*' attention is more stable (less distractible) rather than more flexible than the *lows*' one (Jamieson and Sheehan, 2004; Egner et al., 2005); genetic studies (Presciuttini et al., 2014) have refuted the hypothesis that reduced dopamine catabolism associated with polymorphism of the brain Catechol-O-Methil-Transferase (COMT) may be responsible for the *highs'* attentional abilities, as no difference between *highs* and *lows* has been found in COMT polymorphism (Szekely et al., 2010). Nonetheless, theoretically, higher dopaminergic tone could be sustained in *highs* by other mechanisms such as different receptors density/distribution/sensitivity, dopamine production, and catabolism by the Mono-Amino-Oxidase system.

Since the very beginning of my research activity I have focused my interest on the physiological correlates of hypnotisability rather than on its psychological factors possibly accounting for hypnotic responding (Killeen and Nash, 2003; Dienes et al., 2009; Lynn and Green, 2011). In fact, I considered that the existence of a trait influencing only one aspect of behavior—the proneness to accept suggestions—would be a serious challenge to common sense, all the more that hypnotic performance was considered a consequence of peculiar attentional abilities (Raz, 2005), attention modulates several sensorimotor processes (Woollacott and Shumway-Cook, 2002; Ruff, 2013), cognitive-emotional traits have been associated with peculiar morphological characteristics of cerebellar structures (Picerni et al., 2013), and both the cerebellum and basal ganglia are often involved in non-motor functions (Stoodley, 2012; Leisman and Melillo, 2013; Keren-Happuch et al., 2014).

My intuition was correct. Indeed, I have found hypnotizability-related differences (Carli et al., 2006, 2008; Santarcangelo et al., 2008, 2010; Menzocchi et al., 2010, 2012; Castellani et al., 2011; Scattina et al., 2012) in many aspects of sensori-motor integration in both the absence (Table 1) (Collins and De Luca, 1993; Caratelli et al., 2010; Mecacci et al., 2013) and the presence of suggestions. I have chosen the differences in postural control induced by imagined sensory alteration (Carli et al., 2006; Santarcangelo et al., 2010; Scattina et al., 2012) as the object of this article. I will also show that my physiological approach to the field of hypnotizability allows to suggest that the involuntariness reported by *highs* in their response to sensory suggestions is physiologically sustained and, thus, “real” rather than only subjectively experienced (Santarcangelo et al., 2010).

**Table 1:** **Sensori-motor integration in not hypnotized *highs* not receiving any suggestion**.

**SPINAL REFLEXES DURING LONG LASTING RELAXATION (Carli et al., 2008)**
Habituation of the *soleus* muscle H reflex
Reduction of the F wave frequency of occurrence (right hand abductor digiti minimi^*^)
POSTURAL CONTROL^*^
Greater independence of peripheral information (Santarcangelo et al., 2008)
Larger range of body sway not associated with peripherally-induced correction (Santarcangelo et al., 2008)
Postural behavior similar to *lows* in highly demanding postural tasks (Caratelli et al., 2010; Mecacci et al., 2013)
**WALKING DIRECTION DURING BLINDFOLDED LOCOMOTION (Menzocchi et al., 2010)**
Larger variability in basal conditions
Scarce influence of tonic head rotation
**HAPTIC EXPLORATION**
More accurate angle reproduction (Menzocchi et al., 2012)
More frequent visual recognition of meaningless objects (Castellani et al., 2011)

* *Stabilogram diffusion analysis (Collins and De Luca, 1993) revealed different internal models for postural control in highs and lows during suppression of vision and alteration of the leg proprioceptive information (Santarcangelo et al., 2008). Nonetheless, highs were not less efficient than lows in maintaining balance when postural conditions were highly demanding and required higher engagement of sensory feed-back mechanisms, as occurs on see-saw platforms (Caratelli et al., 2010) and in one legged stance (Mecacci et al., 2013)*.

### Postural effects of imagined sensory alteration

The experience of involuntariness in hypnotic responding has been attributed by neo-dissociative theories to dissociation between experience or executive control and behavior, and by socio-cognitive theories to deep absorption in mental images associated with high expectancy of behavior congruent with the suggestions content (Lynn and Green, 2011).

Different cortical activities have been observed during active, passive and hypnotically-induced movements (Kosslyn et al., 2001; Blakemore et al., 2003; Cardena et al., 2012), and the observed activation of the inferior parietal cortex has been assumed as responsible for perceived involuntariness (Blakemore et al., 2003), whereas physiological automaticity in hypnotic responding has never been demonstrated. I have approached this topic through a number of postural studies (Carli et al., 2006; Santarcangelo et al., 2010; Scattina et al., 2012).

It is well-known that imagined and physically- induced perceptions as well as imagined and real voluntary movements activate the same neural circuits, although to different extent (Jeannerod, 2001; Kosslyn et al., 2001; Moulton and Kosslyn, 2009; Munzert et al., 2009; Hétu et al., 2013). Several observations suggest a more effective activation of these circuits in *highs*.

In fact, only *highs* exhibited a backward body displacement (as expected on the basis of the inertia law) while standing and receiving the instruction to imagine that a carpet was under their feet and was being abruptly pulled forward (Carli et al., 2006). In this study, the expected behavior was not explicitly described by the suggestion script; in addition, participants reported not to have predicted their movement on the basis of pertaining physical laws and to have experienced their backward sway as involuntary. Thus, it appeared unlikely that their movement was triggered by absorption/expectation, as suggested by Kirsch and Lynn (1999), but we did not have any objective evidence of involuntariness.

In another study, during imagery of procedural leg pain (Scattina et al., 2012), that is while imagining that “the surgeon is taking out some pebbles from the left calf, he is using tweezers, he cannot avoid tearing away small pieces of flesh… unfortunately there is no anesthetic…,” only highs exhibited a postural response (larger and faster body sway with respect to rest, displacement of the Center of Pressure (CoP) toward the site opposite to the imagined nociceptive stimulation) congruent with the suggestion administered and reported it as involuntary. Covariate analysis showed that their body displacement was not completely accounted for by the vividness of imagery, absorption in the task and perceived pain intensity; in fact, the CoP displacement toward the opposite side was independent of all these factors. Thus, again our findings challenged the hypothesis that absorption/expectation may be sufficient to account for the observed motor response. Nonetheless, participants may have been aware that pain in one leg induces a displacement toward the other leg and/or remember similar real situations, thus again we had no evidence of involuntariness.

### The vestibulo-spinal reflex: a probe for involuntariness

We designed an experiment to assess whether the perception of involuntariness may be sustained by physiological automaticity (Santarcangelo et al., 2010). In particular, we chose the vestibulo-spinal reflex (VR) evoked by galvanic stimulation of the labyrinth as a “probe for involuntariness.” In fact, the vestibular stimulation induces body sway depending on the head position with respect to the body (Manzoni, 2005; Shaikh et al., 2005), that is in the frontal plane with the participant's head directed forward and in the sagittal plane with the head rotated toward one side. This shift in the direction of body sway depends on the cerebellar integration of vestibular and neck proprioceptive inputs (Kammermeier et al., 2009). In addition, the VR earliest component elicited in any head position is not modulated by expectancy (Guerraz and Day, 2005) and volition (Reynolds, 2010).

We elicited VR in highs and lows with their head directed forward as well as physically and imaginatively rotated toward one side (“… please, imagine that your head is rotated toward the right side… you can see your chin aligned with your shoulder… and feel the tension in your neck muscles… ”) and observed that only the highs' VR earliest component occurred in the sagittal plane for both the real and imagined head rotation, in spite of the similar absorption and vividness of imagery. Given the characteristics of VR, we have to admit that imagery of “rotated head” generated the same neural conditions associated with real head rotation, despite the subjects could not predict which neural circuits should be activated/inhibited and the studied reflex could not be voluntarily controlled. In other words, highs can transform into appropriate neural activations not only the mental images of voluntary movement and of selected perceptions (Jeannerod, 2001; Kosslyn et al., 2001; Moulton and Kosslyn, 2009; Munzert et al., 2009; Hétu et al., 2013), as observed in the general population, but also the images of sensory contexts whose neural correlates cannot be predicted and controlled. The highs' experience of involuntariness may reflect this “automatic” activation of neural circuits enabling these individuals to change their internal model for postural control according to their mental images. This change does not require any dissociative barrier (Dienes et al., 2009; Dell, 2010; Lynn and Green, 2011), is not a consequence of deep absorption/high expectancy (Lynn and Green, 2011), and cannot be assimilated to the preparation of voluntary movements (Custers and Aarts, 2010).

The *highs'* deeper embodiment of the image of rotated head, however, may derive also from their preferential employment of the kinesthetic modality of imagery. In fact, in line with earlier findings (Carli et al., 2007), they more often reported to have “felt the neck muscles tension” rather than to have “seen their chin in line with the shoulder”(Santarcangelo et al., 2010), and the kinesthetic modality is more efficacious than the visual one in the body representation (Shenton et al., 2004).

In contrast to the imagery of rotated head, which consists of an entirely imagined sensory context, the obstructive suggestion of anesthesia, which requires only the ability to attenuate/abolish the perception of a physical stimulation, reduces the amplitude of the VR earliest component in both groups (Santarcangelo et al., 2010), as observed for the suggestion of analgesia during nociceptive stimulation (Santarcangelo et al., 2013). Thus, on the basis of the observed hypnotizability-related capacity to transform mental images into sensori-motor physiological conditions, *highs* may represent the upper extreme of a continuum in the ability to modulate neural conditions through imagery.

## Responses to sensory suggestions and the cerebellum

Impaired performance at cerebellar tests during suggestions of anesthesia had suggested a cerebellar involvement in the motor response to sensory suggestions, but hypnotic relaxation may have influenced the performance (Wallace and Hoyenga, 1981). It has been also shown that overactivity in the cerebellum and in the parietal cortex is associated with the misattribution of actions to an external source (Blakemore et al., 2003), but this report did not consider a possible real, physiologically based involuntariness.

In our study of the imagery of rotated head (Santarcangelo et al., 2010) the embodiment of the mental image of rotated head involuntarily produced is likely to enable the cerebellum to act routinely on the imaginatively constructed sensory information. Alternatively, imagery-triggered top down signals may change the cerebellar internal model according to the mental images' content. In both instances, the perceived involuntariness would be physiologically sustained. In contrast, in the studies on imagery of leg pain (Scattina et al., 2012) and of inertial backward falling (Carli et al., 2006), in which the response to suggestions could be expected and voluntarily controlled (despite the participants' subjective reports), real involuntariness could be sustained by reduced cerebellar inhibition (Groiss and Ugawa, 2013) of motor cortices (Figure 1).

Together with parietal associative areal, the cerebellum is likely to be responsible also for the different internal models (Ito, 2006) for postural control observed in *highs* and *lows* in the absence of suggestions (Santarcangelo et al., 2008). It should be also studied whether cerebellar peculiarities may contribute to the cognitive aspects of hypnotic responding through vestibulo-*coeruleus* and rubro-thalamo-prefrontal pathways (Groiss and Ugawa, 2013).

## Frontiers: hypnotizability and nitric oxide

Studies of the post-occlusion flow-mediated dilation (FMD) of the brachial artery (Green et al., 2014) have indicated greater availability of endothelial nitric oxide (NO) in *highs* during mental stress and nociceptive stimulation (Jambrik et al., 2005). A quite important question is whether, in these individuals, larger NO availability characterizes also the brain vessels. In fact, the brain endothelial NO is responsible for basal vascular tone, interacts with other mediators, and acts as a neurotransmitter after diffusion to the extracellular compartment. Thus, (Figure 1), in the whole brain, endothelial NO could prime cascade processes modulating the neuronal activity in *highs* and *lows* differentially. In addition, in animals NO increases the release of acetylcholine, decreases the dopamine release and increases its metabolism; in humans, larger endothelial NO availability in hypnotizability-related circuits (Hoeft et al., 2012) could account for the *highs'* attentional characteristics (see Presciuttini et al., 2014) and could be involved in the cognitive aspects of hypnotizability.

In the cerebellum NO acts also as a negative regulator of granule cell precursor proliferation, promotes survival and differentiation of these neurons and regulates bidirectional plasticity at parallel fiber-Purkinje neuron synapses (Contestabile, 2012). Thus, in the cerebellum, NO could be responsible for different functional properties. On the whole, *highs* and *lows* may exhibit NO-dependent different activation of sensory and associative regions such as those belonging to the salience and executive circuits, cerebellar inhibition of the motor cortex, internal model for postural control.

In conclusion, our findings allow to include hypnotizability among the individual traits responsible for part of the variability in postural control and indicate that it may be involved in the construction of individual sensorimotor selves. They suggest a key role of the cerebellum in hypnotic responding and go beyond psychological theories by suggesting that the involuntariness in action reported after a few sensori-motor suggestions can be real rather than merely experienced.

### Conflict of interest statement

The author declares that the research was conducted in the absence of any commercial or financial relationships that could be construed as a potential conflict of interest.
